# Genome-Wide Identification of the *CRY* Gene Family in *Solanum tuberosum* and Response to Abiotic Stresses

**DOI:** 10.3390/genes16101234

**Published:** 2025-10-18

**Authors:** Yan Gao, Xueying Yang, Xin Lv, Yuxuan Li, Kuihua Li, Yuliang Gao

**Affiliations:** 1 Agricultural College, Yanbian University, Yanji 133002, China; se252taka851@163.com (Y.G.); 15971689030@163.com (X.Y.); lvxin050423@163.com (X.L.); liyuxuan061124@163.com (Y.L.); 2 Yanbian Academy of Agricultural Sciences, Longjing 133400, China

**Keywords:** abiotic stress, blue-light photoreceptor, *CRY* gene family, *Solanum tuberosum*

## Abstract

Background: Cryptochromes (CRYs) are not only blue-light receptors in plants but also participate in abiotic stress responses, making them essential for plant growth and development. Methods: In this study, the *CRY* gene family in potato (*StCRY*) was identified and analyzed using bioinformatics approaches, and the expression patterns of *StCRY* genes under different abiotic stresses were validated through transcriptome datasets and RT-qPCR analysis. Results: A total of 7 *StCRY* genes were identified, unevenly distributed across 4 chromosomes. The *StCRY* genes exhibit conserved structures, with predicted subcellular localization primarily in the nucleus, cytoplasm, and plastids. Promoter region analysis revealed the significant presence of *cis*-acting elements related to light, plant growth and development, hormones, and stress responses. Phylogenetic analysis classified the *CRY* gene family into three subgroups and identified one pair of collinear genes. *StCRY* genes show a closer evolutionary relationship with tomato, followed by *Arabidopsis thaliana*, and are least related to rice. Transcriptome and RT-qPCR analyses under cold, drought, and salt stresses revealed differential expression among *StCRY* genes: *StCRY3* and *StCRY7* respond positively to cold stress, *StCRY1* and *StCRY5* are upregulated under drought and salt stresses, and *StCRY7* expression is positively correlated with salt stress. Conclusions: Collectively, this study provides a preliminary characterization of the *CRY* gene family in potato and establishes a theoretical foundation for further investigations into the molecular mechanisms of blue-light receptors in abiotic stress responses.

## 1. Introduction

Light is the primary source of energy for plants, influencing photosynthetic reactions and playing a decisive role in plant growth and development [1]. Variations in photoperiod, light intensity, and light quality affect plant growth in different ways, among which light quality is particularly critical [2]. Light quality refers to the wavelength composition of light, that is, its color or spectral distribution. Plants exhibit distinct responses to different wavelengths, such as blue light, red light, and far-red light [3,4]. Light quality transmits signals by activating specific photoreceptors in plants and subsequently modulates intracellular signaling pathways, thereby influencing enzyme activity and hormonal changes, which ultimately affect plant growth and development, secondary metabolism, and yield quality [5,6]. In practical production, supplementary red and blue light is commonly applied to optimize plant growth and improve the quality of horticultural products [7,8].

To better adapt to the light conditions of their environment, plants have evolved a variety of photoreceptors during evolution, including phytochromes, the UV-B receptor UVR8, and cryptochromes, among others [9,10,11]. Blue-light photoreceptors mainly consist of cryptochromes (CRYs) and phototropins [12]. *CRYs* regulate multiple key stages of plant growth and development, such as seedling de-etiolation, photosynthesis, hypocotyl elongation, and responses to abiotic stresses [13,14,15]. Cryptochromes are primarily composed of CRYs and CRY-DASH [16,17]. It is generally believed that *CRY1* acts as the major factor in blue-light-dependent photomorphogenesis, while *CRY2* is mainly involved in photoperiodic regulation of flowering time, with functional redundancy between the two in the regulation of flowering [13]. The CRY-DASH protein contains only an N-terminal domain, and its precise role in plants remains unclear. Some studies suggest that it can directly bind DNA and RNA and mediate light-dependent repair of UV-damaged DNA [15,16,17].

During plant growth and development, AtCRY1 directly interacts with AUX/IAA to inhibit the binding of TIR1 and AUX/IAA, thereby preventing the degradation of AUX/IAA. This suppression leads to inhibited hypocotyl elongation and enhanced anthocyanin accumulation. Moreover, *cry1* mutants exhibit delayed flowering under various light conditions [18]. *AtCRY2* primarily suppresses the transcription of root cell division genes in darkness, while blue light alleviates this inhibitory effect [19]. In tomato, *cry1a* mutants display relatively taller plants and increased fruit set, whereas overexpression produces the opposite phenotype [20]. In rice, *OsCRY1s* inhibit coleoptile and leaf growth under blue light and mediate the de-etiolation response, while *OsCRY2* is involved in promoting flowering time [21].

*CRYs* play important roles in plant responses to temperature, drought, and salt stresses [22,23]. *CRY1* regulates plant thermotolerance through the Constitutive Photomorphogenic 1 (*COP1*)–Elongated Hypocotyl 5 (*HY5*) module. Under normal- and high-temperature dark conditions, COP1 mediates the degradation of HY5 protein, thereby releasing the repression of heat shock transcription factors (HSFs) by HY5; as a result, HSFs are highly expressed in response to heat stress in darkness. Under high-temperature blue light conditions, elevated temperature inhibits the activity of CRY1-associated protein kinases (PPKs), leading to a reduction in blue-light-dependent phosphorylation of *CRY1*, weakened CRY1–COP1 interactions, increased *COP1* activity, and decreased *HY5* abundance, ultimately resulting in enhanced expression of *HSFs* [24]. The *CRY2-COP1-HY5* module in *Arabidopsis* positively regulates plant freezing tolerance by activating a subset of cold-responsive genes through BBX7/8 transcription factors, in a manner independent of CBF/DREB2 pathways [25]. In addition, the blue-light receptor *CRY* integrates abscisic acid (ABA) and jasmonic acid (JA) signaling into the plant salt stress response by interacting with GBF1 and other regulatory factors [26].

Potato (*Solanum tuberosum*), a member of the *Solanaceae* family, is an annual herbaceous plant widely cultivated worldwide and ranks as the fourth most important food crop [27]. Its tubers are rich in starch, proteins, and various vitamins and minerals [28]. In recent years, with increasing research on its nutritional value, the applications of potato in food processing, animal feed, and biofuel production have attracted growing attention [29]. Studies indicate that potato not only contributes to improving human health but also plays a vital role in agricultural economies [30,31]. The present study identifies and analyzes the *CRY* gene family in potato using bioinformatics approaches and further characterizes them through transcriptome datasets under multiple abiotic stresses and RT-qPCR analyses. These findings provide a theoretical foundation for future research on potato yield improvement.

## 2. Materials and Methods

### 2.1. Plant Materials and Abiotic Stress Treatments

The potato cultivar daxiyang was selected as the experimental material. Potato tubers were planted in soil (nutrient soil:vermiculite = 3:1) and incubated for 30 days under controlled conditions of 24 °C, a light intensity of 2000 Lx, and a 16 h light/8 h dark photoperiod. Subsequently, plants with five leaves and a single shoot apical meristem were selected for further experiments. The plants were subjected to cold treatment (4 °C), salt stress (80 mM NaCl) [32], and drought stress (20% PEG600) [33], and samples were collected at 0 h, 1 h, 3 h, 6 h, 12 h, and 24 h [34,35,36]. All treatments were performed with three biological replicates. The samples were immediately frozen in liquid nitrogen and stored at −80 °C for subsequent analyses.

### 2.2. Identification of StCRY Gene Family Members

The potato genome (*Solanum tuberosum* v6.1), protein sequence files, and genome annotation files were downloaded from the Phytozome website (https://phytozome-next.jgi.doe.gov/, accessed on 10 July 2025). The *CRY* hidden Markov model (HMM, PF00875; DNA_photolyase) was obtained from the Pfam database (https://www.ebi.ac.uk/interpro/, accessed on 10 July 2025), and HMMER v3.3 software was used to search the potato protein sequence files. Additionally, *A. thaliana* CRY protein sequences were downloaded from Phytozome and used as queries for BLASTp searches against the potato protein sequences to identify candidate genes. The candidate genes obtained were further verified for domain composition using SMART (http://smart.embl.de/, accessed on 10 July 2025), NCBI Conserved Domain Database (https://www.ncbi.nlm.nih.gov/cdd/, accessed on 10 July 2025), and Pfam, resulting in the final identification of confirmed potato *CRY* genes.

### 2.3. Chromosomal Localization and Physicochemical Properties Analysis of StCRY Genes

Based on the potato genome annotation file, the lengths of the 12 chromosomes and the chromosomal positions of the *StCRY* genes were obtained. TBtools (v2.333) was used to generate a chromosomal localization map of *StCRY* genes, displaying the positions and relative distances of all members of the *StCRY* gene family on the chromosomes [37]. The protein sequences of *StCRY* genes were submitted to the ProtParam website (https://web.expasy.org/protparam/, accessed on 10 July 2025) for physicochemical property analysis. Subcellular localization of StCRY proteins was predicted using the Cell-PLoc-2.0 website (http://www.csbio.sjtu.edu.cn/bioinf/plant-multi/, accessed on 10 July 2025).

### 2.4. Analysis of Conserved Domains, Promoter Regions, and Gene Structures of StCRY Proteins

Conserved motif analysis of StCRY proteins was performed using the MEME software (https://web.mit.edu/meme_v4.11.4/share/doc/meme-format.html, accessed on 10 July 2025) with the minimum motif width set to 6 and the maximum width set to 100. MEME analysis data were extracted using a Perl script. Gene structure annotation information for *CRY* genes was obtained from the potato genome annotation file, and TBtools was used to visualize motifs, and gene structures.

For promoter analysis, 2 kb upstream sequences of all *StCRY* genes were extracted from the potato genome and annotation files. These sequences were submitted to the PlantCARE database for *cis*-acting element analysis, and the results were visualized using ggplot2 in R (v4.02).

### 2.5. Phylogenetic and Synteny Analysis of the StCRY Gene Family

Genome files and CRY family protein sequences of *Oryza sativa*, *A. thaliana*, and *Solanum lycopersicum* were downloaded from the Phytozome database and the Sol Genomics Network website(https://solgenomics.net/, accessed on 15 July 2025). A phylogenetic tree was constructed using MEGA software (version: X) [38], with sequence alignment performed by Clustal W and the Maximum Likelihood method applied for tree construction. The resulting phylogenetic tree was visualized and beautified using Evolview v2 (https://evolgenius.info/evolview-v2/, accessed on 15 July 2025).

For duplication analysis, the potato whole-genome sequences were self-compared using TBtools to identify duplication events among StCRY family members. Based on protein sequences and genome annotation files of rice, *Arabidopsis*, tomato, and potato, MCScanX was used to analyze the genomes and determine the collinearity relationships of *StCRY* genes between potato and rice, *Arabidopsis*, and tomato.

### 2.6. Protein–Protein Interaction and miRNA Prediction of StCRY

To construct the miRNA-gene interaction network, we conducted an analysis of miRNA targeting *StCRY* genes on the psRNATarget website (https://www.zhaolab.org/psRNATarget/, accessed on 15 July 2025). Protein–protein interaction (PPI) predictions among potato CRY proteins were performed using STRING (https://string-db.org/, accessed on 15 July 2025), and the results were visualized in R. To construct the miRNA-gene interaction network, miRNAs targeting *StCRY* genes were analyzed using the psRNATarget website (https://www.zhaolab.org/psRNATarget/, accessed on 17 July 2025).

### 2.7. Expression Analysis of StCRY Gene Family Members Based on Abiotic Stress Transcriptome Data

Transcriptome data of potato under cold, drought, and salt stress were downloaded from the NCBI GEO database. Salmon was used to map the transcriptome data and extract expression levels of all members of the *CRY* gene family [39]. Heatmaps were generated in R to visualize the results, and the expression changes in CRY family members under cold stress were analyzed.

### 2.8. Gene Ontology Enrichment Analysis

Gene Ontology (GO) enrichment analysis of the *StCRY* gene family was performed as follows. First, functional annotation of all *StCRY* family members was conducted using eggNOG-mapper (http://eggnog-mapper.embl.de/, accessed on 17 July 2025). Based on the annotation results, GO enrichment analysis was carried out to identify significantly overrepresented biological processes, molecular functions, and cellular components within the gene family. Finally, the enrichment results were visualized using R, enabling a clear representation of GO term distributions and their significance.

### 2.9. Quantitative Real-Time PCR (RT-qPCR) Analysis

Total RNA was extracted from potato leaves using Plant RNA Rapid Extract Kit (Coolaber, Beijing, China) and reverse transcribed into cDNA using NotionScript qPCR First-strand cDNA Synthesis Mix (Coolaber, Beijing, China). Fluorescent quantitative analyzed using 2× SYBR Green qPCR Mix (Coolaber, Beijing, China) for RT-qPCR. Primer sequences are provided in Table S3. The RT-qPCR reaction system and conditions were adjusted based on the methodology described by. Relative gene expression levels were calculated using the 2^−∆∆Ct^ method [40]. The *β-actin* gene was used as a standardized internal reference and 3 biological replicates were set up for each sample [41].

### 2.10. Statistical Analysis

Experimental results are presented as the mean ± standard error, based on at least three independent biological replicates. Statistical analyses were performed using two-way analysis of variance (ANOVA) in SPSS version 25.0 (SPSS Inc., Chicago, IL, USA) to determine *p*-values, with *p* < 0.05 considered statistically significant. Data visualization was carried out using GraphPad Prism 9.

## 3. Results

### 3.1. Identification and Physicochemical Property Analysis of StCRY Gene Family Members

**Figure 1:**
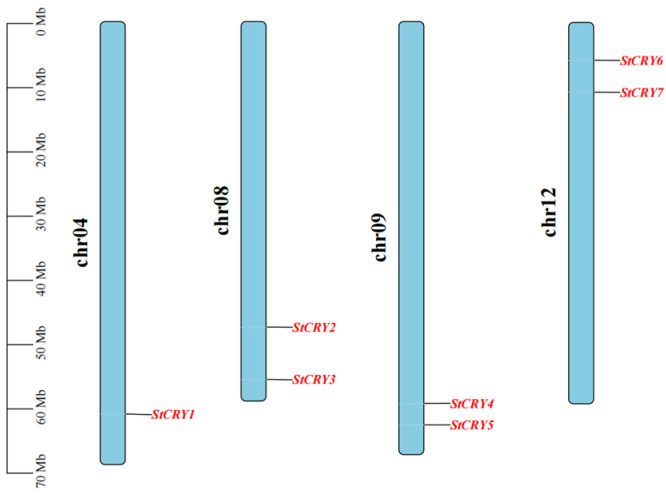
Chromosomal distribution of *StCRY* gene family members in potato. Blue vertical bars represent potato chromosomes, with the scale on the left indicating chromosome length (Mb).

First, we identified 6 *StCRY* genes by aligning potato protein sequences with *Arabidopsis* CRY protein sequences. We then performed a genome-wide search of potato using the known CRY HMM profile (PF00875), which led to the identification of 7 *StCRY* genes in total. By integrating the results from both approaches and conducting transcriptome-based redundancy removal, domain analysis, and sequence alignment, we ultimately confirmed seven *StCRY* genes. These seven genes are distributed across four chromosomes (Figure 1): chr4 (*StCRY1*), chr8 (*StCRY2* and *StCRY3*), chr9 (*StCRY4* and *StCRY5*), and chr12 (*StCRY6* and *StCRY7*). Based on their chromosomal positions, we named the genes *StCRY1~7.* The encoded proteins range from 385 to 679 amino acids in length, with predicted isoelectric points between 5.23 and 9.23. All proteins have GRAVY values below zero, indicating that they are hydrophilic. Subcellular localization prediction suggests that StCRY proteins are mainly localized in the nucleus, cytoplasm, and plastids, implying that *CRY* genes may perform diverse functions (Table S1).

### 3.2. Phylogenetic Analysis of the CRY Gene Family

**Figure 2:**
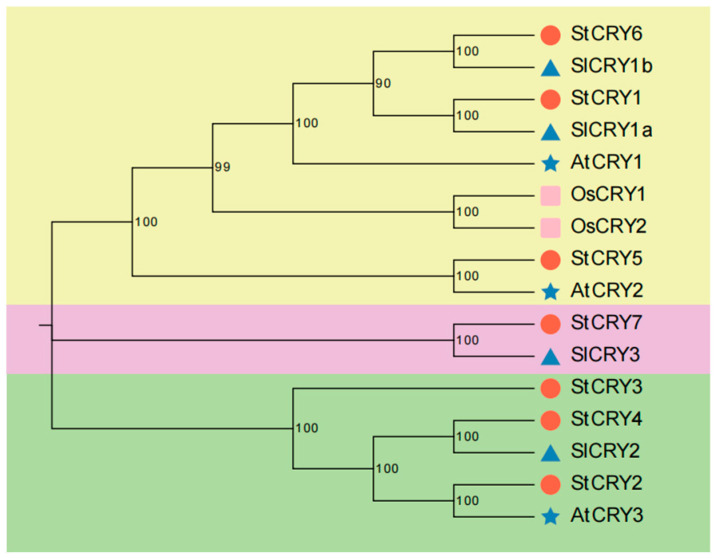
Phylogenetic tree of *CRY* gene family members from potato (*Solanum tuberosum*, St), tomato (*Solanum*. *lycopersicum*, Sl), *A*. *thaliana* (At), and rice (*O*. *sativa*, Os). Red circles indicate potato, blue triangles indicate tomato, blue stars indicate *Arabidopsis*, and pink squares indicate rice. Bootstrap values are shown at each node.

To further investigate the evolutionary relationships of *StCRY* genes, we retrieved CRY protein sequences from rice, *Arabidopsis*, and tomato to construct a phylogenetic tree (Figure 2). Our results classified the *CRY* genes into three major groups: *StCRY1*, *StCRY5*, and *StCRY6* were assigned to the first group; *StCRY7* formed the second group; and *StCRY2*, *StCRY3*, and *StCRY4* clustered into the third group. Phylogenetic analysis further revealed that *StCRY7* and *SlCRY3* belong to the same subgroup. These findings indicate that *CRY* genes in potato are more closely related to those in tomato, while rice *CRY* genes are more distantly related, suggesting that *CRY* genes may perform similar functions in potato and tomato.

### 3.3. Analysis of Conserved Motifs and Gene Structures of StCRY

**Figure 3:**
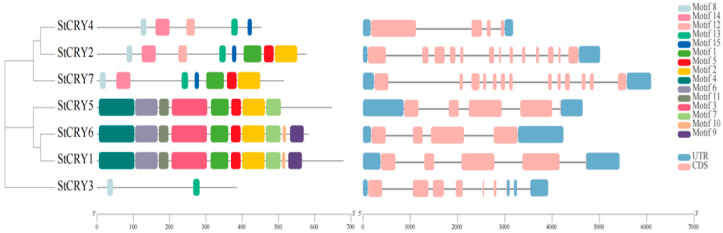
Conserved motifs and gene structures of *StCRY* family members. The left panel shows the distribution of conserved motifs in StCRY proteins, with different colored boxes representing distinct motifs (Motif 1–15). The right panel illustrates the exon–intron structures of *StCRY* genes, where pink boxes indicate coding sequences (CDS), blue boxes represent untranslated regions (UTRs), and black lines denote introns.

We analyzed and visualized the conserved motifs of StCRY proteins using MEME and TBtools (Figure 3). Based on motif analysis, we classified the *StCRY* genes into three groups: the first group includes *StCRY2*, *StCRY4*, and *StCRY7*; the second group comprises *StCRY1*, *StCRY5*, and *StCRY6*; and the third group contains *StCRY3*. All genes in the first group contain motifs 8, 14, 13, and 15. The second group primarily includes motifs 4, 5, 11, 3, 1, 5, 2, and 7. The third group mainly consists of motifs 8 and 13. Proteins encoded by genes within the same group exhibit highly similar types and numbers of conserved motifs, indicating a strong conservation of motif composition within each subgroup.

We visualized the gene structures of *StCRY* based on the annotation files (Figure 3). We found that the number of exons and introns in *StCRY* genes ranges from 4 to 13. Both *StCRY2* and *StCRY7* contain 13 exons and introns. *StCRY4* and *StCRY6* each contain 4 exons, while *StCRY1* and *StCRY5* each contain 5 exons. Notably, the 3′UTR of *StCRY3* contains three discontinuous segments. Although the coding sequence lengths are similar, the variation in exon and intron numbers during evolution leads to significant differences in the full gene lengths. This structural diversity may contribute to functional variability and provides valuable reference for further studies on the evolutionary relationships of the *StCRY* gene family in potato.

### 3.4. Cis-Element Analysis of StCRY Promoters

**Figure 4:**
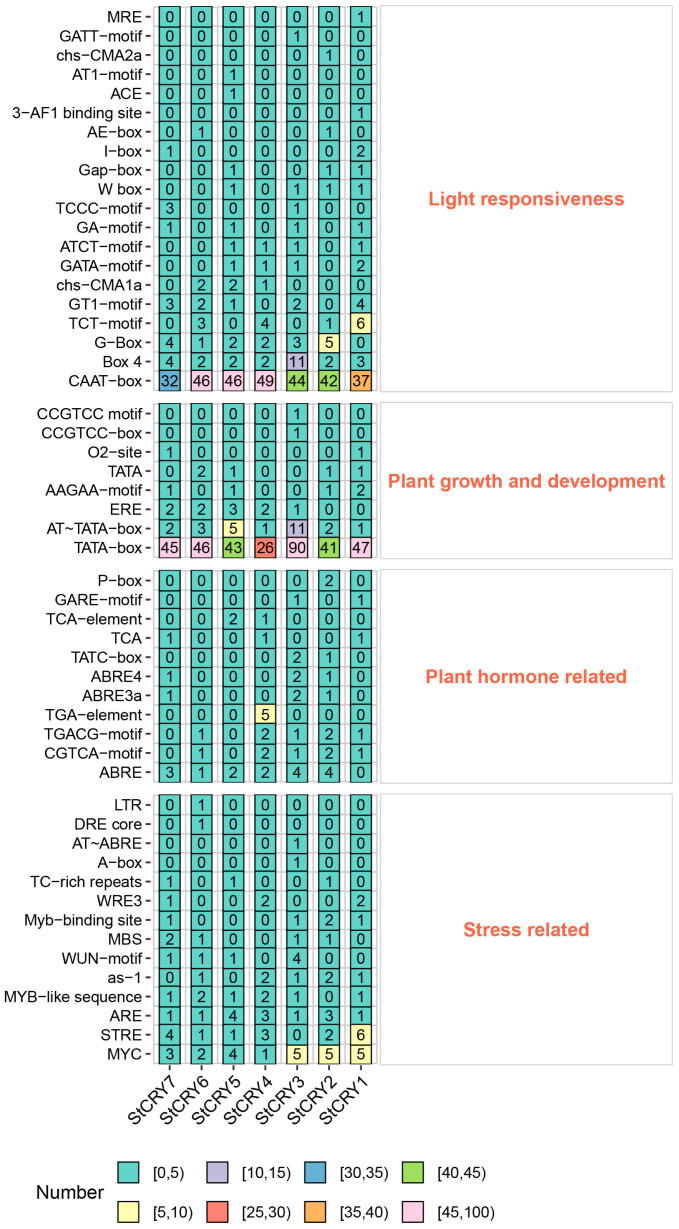
*Cis*-acting elements in *StCRY* promoters. The number of each element is indicated within the corresponding grid, and the background color of the grids represents the abundance of the elements. The red labels denote the four different categories of promoter *cis*-acting elements.

We analyzed the promoter regions of *StCRY* genes and classified their *cis*-acting elements into four categories (Figure 4): light-responsive elements, elements related to plant growth and development, hormone-responsive elements, and stress-responsive elements. Among the light-responsive elements, we found that all genes except *StCRY1* contain G-BOX elements, with *StCRY2* containing five copies. *StCRY3* and *StCRY7* lack T-motif elements, whereas *StCRY1* contains six copies. Although *StCRY* genes function as blue-light receptors, not all genes possess the corresponding *cis*-elements. All genes from *StCRY3* to *StCRY7* contain ERE elements, suggesting that these genes may participate in regulating plant senescence. Various hormone-responsive elements are present in *StCRY* genes, including ABA-responsive ABRE elements (*StCRY2*–*StCRY7*), methyl jasmonate-responsive CGTCA-motif and TGACG-motif elements (*StCRY1*, *StCRY2*, *StCRY3*, *StCRY4*, and *StCRY6*), and the auxin-responsive TGA-element (*StCRY4*). Additionally, all *StCRY* genes contain MYC, MYB, and ARE elements, indicating potential roles in stress responses.

### 3.5. Tandem Gene Duplication and Segmental Gene Duplication of StCRY

**Figure 5:**
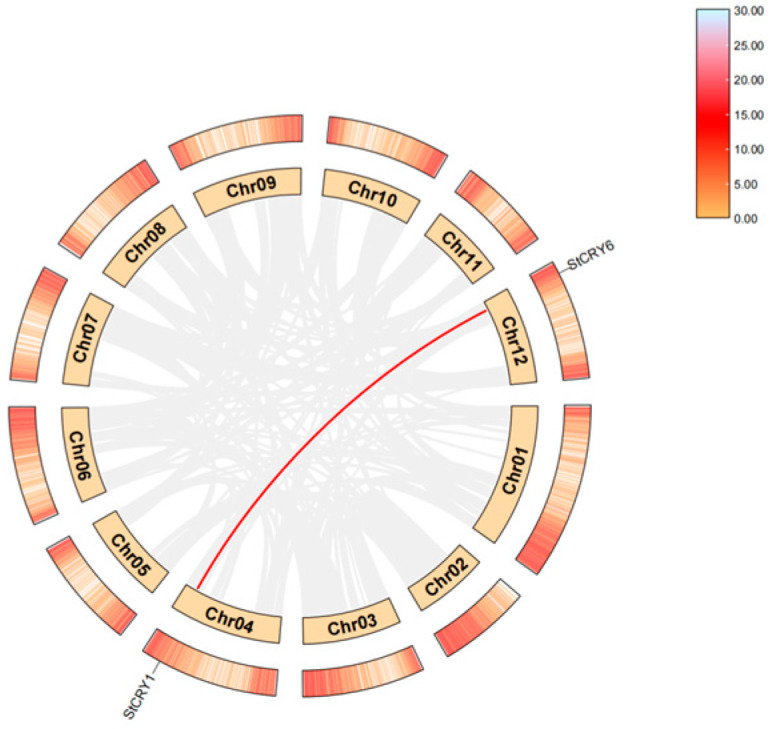
Collinearity among *StCRY* homologous genes. Gray lines in the background represent the collinearity of genes across the whole genome, while red lines connect *StCRY* gene pairs with collinear relationships. The outer red rectangles indicate the gene density across the genome.

The Ka/Ks ratio, which compares nonsynonymous substitutions (Ka) to synonymous substitutions (Ks), is a key indicator of selective pressure on protein-coding genes and holds significant importance in evolutionary analysis [42]. In this study, we analyzed the Ka/Ks values of the coding regions of *StCRY* genes and found that all *StCRY* members exhibited Ka/Ks ratios below 1, indicating strong purifying selection during their evolutionary history and suggesting potential functional conservation among these genes (Table S2). Given that *StCRY* genes are unevenly distributed across four chromosomes (Figure 5), we conducted segmental duplication analysis and identified a segmental duplication between *StCRY1* and *StCRY6*. Such duplications may promote genetic diversity within the genome by altering gene copy number and structural variation, thereby influencing growth and development, trait expression, genetic variation, and other vital biological processes.

**Figure 6:**
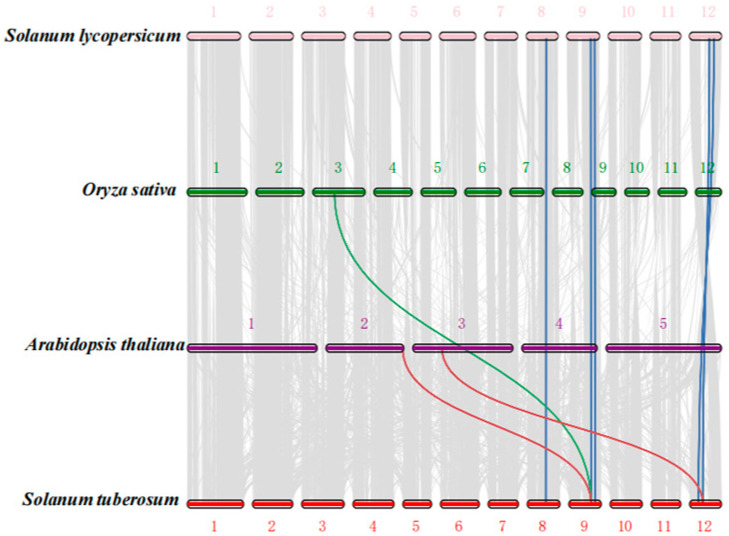
Comparative collinearity of *StCRY* genes across multiple plant species. Gray lines in the background connect collinear genes across the genomes of different species. Blue lines indicate collinear *StCRY* gene pairs between potato and tomato, green lines indicate collinear pairs between potato and rice, and red lines indicate collinear pairs between potato and *Arabidopsis*.

To further investigate the genetic relationships among *CRY* family members, we performed interspecies collinearity analysis using the genomes of *Arabidopsis*, rice, and tomato (Figure 6). We found two collinear gene pairs between *Arabidopsis* and potato, one pair between rice and potato, and five pairs between tomato and potato. These results suggest a closer evolutionary relationship among species within the same family, with genes likely sharing similar functions. Therefore, we infer that these duplication events may have occurred during the evolutionary history of the *CRY* gene family.

### 3.6. GO Enrichment Analysis of StCRY Genes

**Figure 7:**
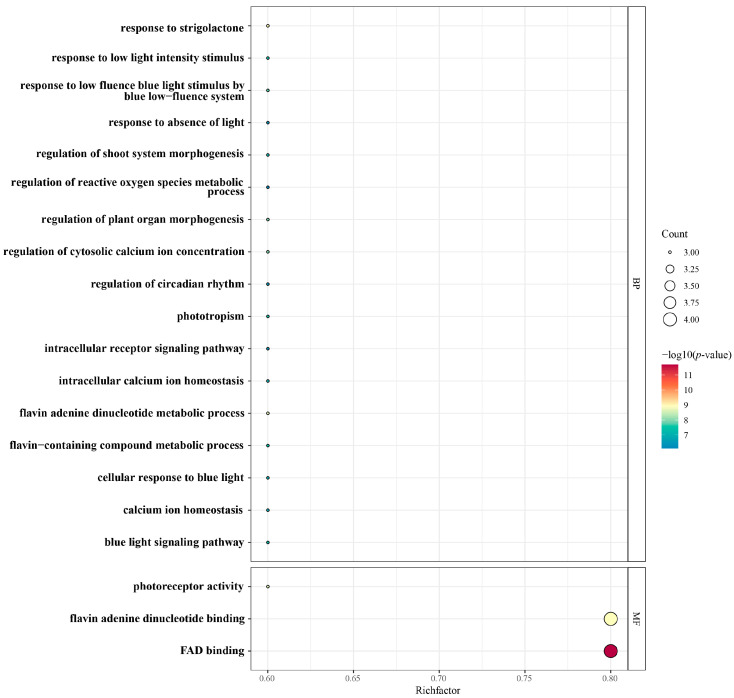
GO enrichment of *StCRY* genes. The horizontal axis shows the proportion of genes associated with each GO term. Circle size represents the number of genes, and color indicates the −log10 (*p*-value).

We performed Gene Ontology (GO) functional annotation to classify *StCRY* genes (Figure 7) into three categories: biological process, cellular component, and molecular function. Since the proportion of cellular component annotations was the lowest, the majority of annotations were concentrated in biological process and molecular function. We ranked the results by *p*-value and selected the top 20 categories for visualization. Our analysis revealed that the annotations were primarily enriched in biological process and molecular function, with molecular function representing the largest proportion. Specifically, *StCRY* genes are mainly involved in FAD binding (GO:0071949, GO:0050660) and photoreceptor activity (GO:0009881). In terms of biological processes, *StCRY* genes are predominantly associated with blue-light response, light signaling pathways, phototropism, photoperiod regulation, energy metabolism, and cell development.

### 3.7. Prediction of miRNA-StCRY Gene Interaction Network

**Figure 8:**
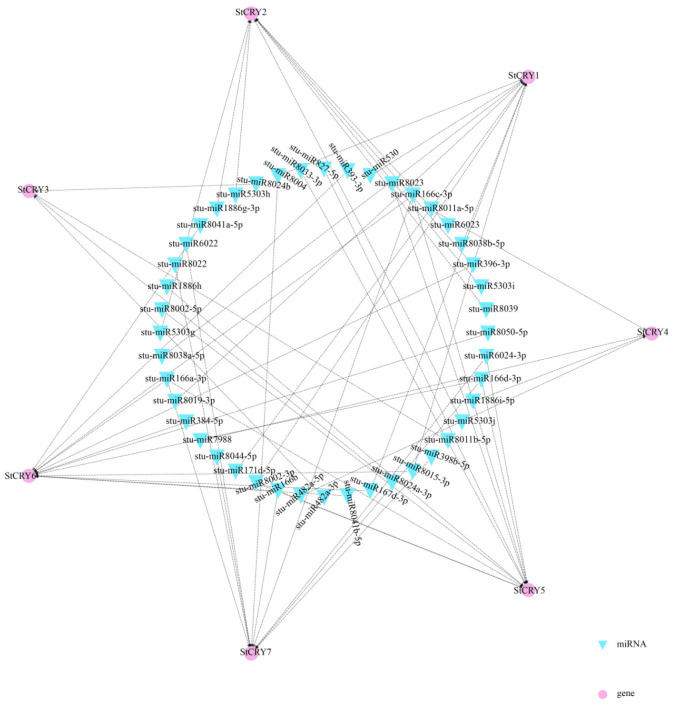
Potential regulation of *StCRY* genes by miRNAs. Purple circles represent *StCRY* genes, and blue triangles represent miRNAs. Black dashed lines connect miRNAs and *StCRY* genes showing regulatory correlations.

To investigate the potential regulatory roles of miRNAs on *StCRY* genes, we predicted miRNA targets using the psRNATarget website. Our results indicated that multiple *StCRY* genes correspond to multiple miRNAs (Figure 8). For example, members of the miR166 family (such as stu-miR166a-3p, stu-miR166b, stu-miR166c-3p, and stu-miR166d-3p) were predicted to target *StCRY7*, *StCRY5*, and *StCRY6*, demonstrating a one-to-many relationship in which multiple miRNAs share the same target genes. Additionally, stu-miR396-3p was predicted to simultaneously target *StCRY6* and *StCRY2*, illustrating a single miRNA regulating multiple genes. The relationships between miRNAs and *StCRY* genes are complex and highly specific, suggesting that these miRNAs may participate in regulating multiple physiological processes in plants.

### 3.8. Prediction of Protein–Protein Interactions of StCRY

**Figure 9:**
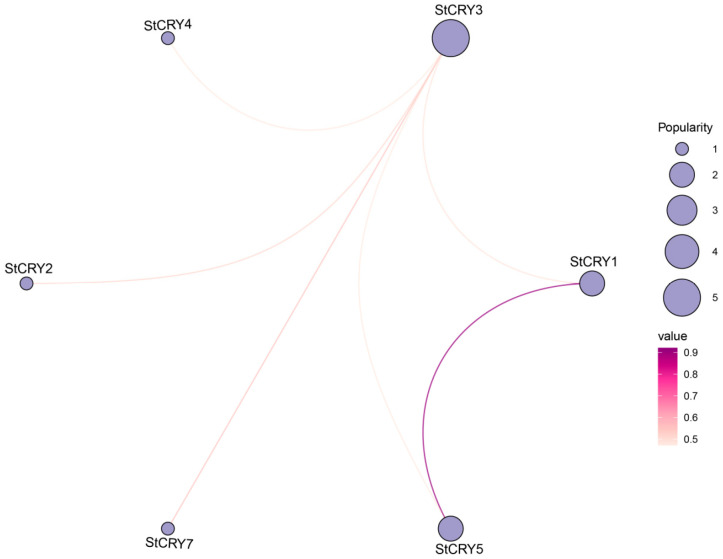
Interaction network of StCRY proteins. Purple circles represent gene popularity, with circle size indicating the level of popularity. Lines connect genes showing correlations, and gradient colors indicate the strength of these correlations.

Proteins are the primary executors of gene function, and analysis of their interaction networks is an important approach to elucidate biological mechanisms. To further investigate the biological functions of StCRY proteins, we constructed a protein–protein interaction network based on the STRING database (Figure 9). Our analysis revealed a significant interaction between StCRY1 and StCRY5, suggesting a potential functional synergy between these two proteins. Notably, StCRY3 was identified as a key hub protein, potentially interacting with multiple family members, including StCRY1, StCRY2, StCRY4, StCRY5, and StCRY7. This indicates that StCRY3 may play a central regulatory role in mediating light signaling pathways.

### 3.9. Expression Analysis of StCRY Genes Under Abiotic Stress

**Figure 10:**
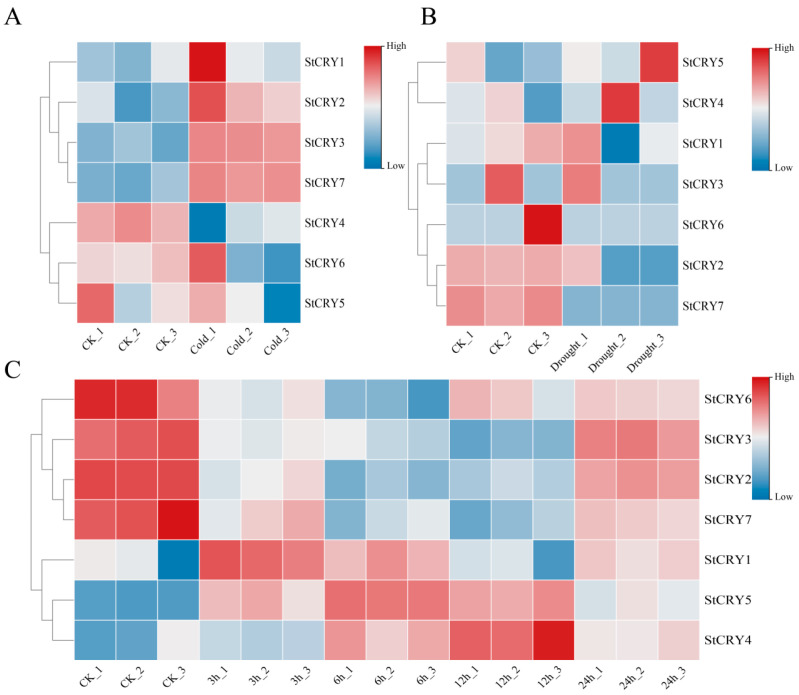
Heatmap of *StCRY* genes expression in potato under abiotic stresses. (**A**) Cold stress (CK: control; Cold: cold treatment). (**B**) Drought stress (CK: control; Drought: drought treatment). (**C**) Salt stress (CK: control; 3 h, 6 h, 12 h, and 24 h represent different time points of salt treatment). _1, _2, and _3 indicate three biological replicates.

As the fourth largest food crop worldwide, potato growth and development are significantly inhibited by abiotic stresses such as temperature extremes, drought, and salinity, which directly affect tuber yield and quality and pose potential threats to food security. We analyzed the expression of *StCRY* genes using publicly available transcriptomes under cold stress (PRJNA753086), drought stress (PRJNA998742), and salt stress (PRJNA882516) from NCBI (Figure 10). Under cold stress (Figure 10A), the expression levels of *StCRY2*, *StCRY3*, and *StCRY7* were upregulated, whereas *StCRY4* and *StCRY6* were significantly downregulated. During drought stress (Figure 10B), *StCRY1*, *StCRY2*, and *StCRY7* showed decreased expression. In the salt stress transcriptome (Figure 10C), the expression of *StCRY2*, *StCRY3*, *StCRY6*, and *StCRY7* decreased at 6 h and 12 h but increased at 24 h, with *StCRY3* showing a pronounced increase at 24 h. *StCRY1*, *StCRY4*, and *StCRY5* exhibited an initial increase followed by a decrease. These genes may serve as potential candidates for studying abiotic stress responses in potato.

**Figure 11:**
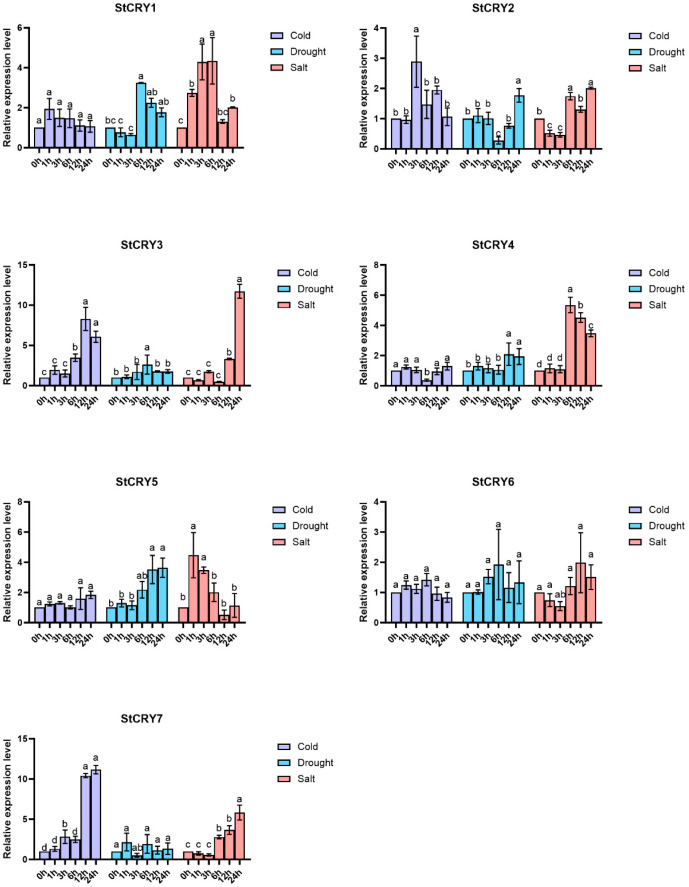
RT-qPCR validation of *StCRY* gene expression under abiotic stresses in potato. Purple bars represent cold stress, blue bars represent drought stress, and red bars represent salt stress, with samples collected at 0 h, 1 h, 3 h, 6 h, 12 h, and 24 h. Different lowercase letters indicate significant differences (*p* < 0.05).

In this study, we subjected potato plants to cold, drought, and salt stresses and analyzed the expression levels of *StCRY* genes at different time points (0 h, 1 h, 3 h, 6 h, 12 h, and 24 h) using RT-qPCR (Figure 11). Under cold stress, *StCRY3* and *StCRY7* showed a marked upregulation compared to 0 h, with *StCRY3* exhibiting approximately a 10-fold increase at 12 h and 24 h. Under drought stress, *StCRY1* displayed an upregulation at 6 h, with expression levels roughly three times higher than at 0 h. Under salt stress, *StCRY1* and *StCRY5* were upregulated at 1 h and 3 h but decreased significantly at 12 h and 24 h. Notably, *StCRY7* showed an approximately 8-fold increase in expression at 24 h compared to 0 h.

## 4. Discussion

Potato, as a globally important food crop, is rich in essential nutrients required by the human body, and its yield plays a critical role in ensuring food security and promoting economic development [30]. Light conditions are one of the key factors affecting potato yield, with different light qualities and photoperiods significantly influencing plant growth and productivity [43,44]. Studies have shown that blue light can enhance plant height, fresh weight, and dry weight of potato plants, as well as promote tuber enlargement [45]. In practical cultivation, an appropriate red-to-blue light ratio can optimize potato growth and yield, while reducing the red-to-blue light ratio promotes tuber expansion, thereby increasing overall yield [44]. Therefore, the rational management of light conditions is of significant importance for improving both the yield and quality of potatoes.

Based on GO annotation classifications, CRY proteins serve as key blue-light photoreceptors in plants and play critical roles in regulating plant growth and development. In this study, seven *StCRY* genes were identified through bioinformatics analyses, which were unevenly distributed across four chromosomes. Motif analysis grouped these genes into three major classes, with members within the same class exhibiting similar motifs and gene structures, suggesting potential functional similarity. It is noteworthy that the phylogenetic and motif analyses produced slightly different groupings of *StCRY* genes. For example, *StCRY7* clusters with *StCRY2* and *StCRY4* in the motif analysis but forms an independent group in the phylogenetic tree. This suggests that despite evolutionary divergence, these genes may retain conserved functional motifs, indicating potential functional similarity. The number of *CRY* genes varies among different species, with strong collinearity observed between potato and tomato, followed by *Arabidopsis* and then rice. The Ka/Ks ratios of *StCRY* genes were all less than 1, indicating that these genes may have undergone purifying selection and segmental duplication throughout evolution. These tandem and segmental duplications likely facilitated the expansion of new members and functions within the *CRY* gene family in the potato genome, thereby enhancing the environmental adaptability of potato.

*CRY* receptors exist as monomers in the dark and undergo conformational changes and oligomerization upon blue-light absorption, thereby altering their interactions with other proteins and influencing plant growth and development. *CRY* receptors can be activated not only by blue light but also function in the dark [46,47]. The G-BOX is an important light-responsive *cis*-element; in this study, only *StCRY1* lacked this element, indirectly suggesting that *StCRY1* may play a significant role under dark conditions.

Due to their sessile growth habit, plants must constantly perceive changes in their surrounding environment. Recent studies have shown that plants adjust their responses to abiotic stress signals based on environmental light cues [48,49]. In this study, transcriptome analyses under cold, drought, and salt stress were conducted to examine *StCRY* gene expression. *CRY* genes exhibited differential responses under these stresses, indicating their involvement in environmental adaptation. These results indicate that potato responds to environmental stresses in part through the blue-light receptor *StCRY*. It is worth noting that some discrepancies were observed between the transcriptome heatmap (Figure 10) and the RT-qPCR results (Figure 11), such as *StCRY1* and *StCRY6* under cold stress. This may be due to the higher sensitivity and broader dynamic range of RNA-seq compared with RT-qPCR, and the differences in sampling time points may also contribute to the observed expression variations. Nevertheless, the overall expression trends are consistent, supporting the reliability of our findings. These results suggest that StCRY proteins may similarly integrate light cues with hormonal signaling (ABA/JA) and the COP1–HY5 module to orchestrate adaptive responses to environmental stresses in potato, consistent with mechanisms reported in *Arabidopsis* [24,25].

MiR166 is a highly conserved microRNA widely present in plants, capable of regulating the expression of HD-ZIP III family transcription factors to modulate plant growth, development, and stress responses [50,51]. In alfalfa (*Medicago sativa*), miR166 expression is downregulated after 6 h of drought stress but upregulated at 24 h [52]. During salt stress, reduced miR166 expression leads to increased PHB expression, thereby affecting root development [53]. MiR166 also plays a significant role in soybean responses to cold, drought, and salt stress [54]. Through miRNA target prediction, *StCRY7*, *StCRY5*, and *StCRY6* were identified as potential interaction partners of miR166, suggesting their involvement in stress response regulation.

Protein–protein interaction predictions identified *StCRY3* as a central hub gene, potentially interacting with multiple other StCRY proteins. Analysis of its promoter region revealed that *StCRY3* contains 90 TATA-box elements, the highest number among the *StCRY* genes, suggesting a potentially strong transcriptional efficiency. Additionally, the promoter sequence of *StCRY3* harbors multiple *cis*-elements responsive to abiotic stress, including three G-Box elements, one W-box, one ERE, four ABREs, five MYC, and three MYB elements, among others. Consistently, its expression was significantly upregulated under both cold and salt stress conditions. These findings highlight the potential of *StCRY* genes not only in stress adaptation but also as candidate genes for breeding programs aimed at improving potato resilience and productivity. Future studies could focus on functional validation and the incorporation of specific *StCRY* genes into breeding strategies to enhance stress tolerance.

## 5. Conclusions

Potato, as a multifunctional crop, is rich in starch as well as various vitamins and minerals. Its advantages include efficient water use and relatively low environmental dependence, making it suitable for cultivation in resource-limited areas. In this study, 7 *StCRY* genes were identified from the potato genome, unevenly distributed across four chromosomes and exhibiting conserved structures during evolution. Their promoter sequences contain a variety of *cis*-regulatory elements related to light, growth and development, hormones, and stress responses. GO enrichment analysis indicated that *StCRY* genes respond to blue light. Gene expression profiling revealed that *StCRY3* and *StCRY7* showed significant upregulation at 12 h and 24 h under cold and salt stress, while *StCRY1* exhibited a notable increase under drought stress, suggesting that abiotic stresses can influence plant blue-light receptors and thereby affect growth and development. These findings provide a theoretical foundation for further studies on the roles of *StCRY* genes in potato growth, development, and responses to abiotic stress.

## Data Availability

The original contributions presented in this study are included in the article/Supplementary Materials. Further inquiries can be directed to the corresponding authors.

## Supplementary Materials

The following supporting information can be downloaded at https://www.mdpi.com/article/10.3390/genes16101234/s1. Table S1: Physicochemical characteristics of *StCRY*; Table S2: Ka/Ks analysis of *StCRY*; Table S3: Primers of *StCRY*.
