# Supplementary material for: Genome-Wide Identification of the CRY Gene Family in Solanum tuberosum and Response to Abiotic Stresses
Source: Genes (Basel). 2025 Oct 18;16(10):1234. doi: 10.3390/genes16101234 (PMC12562852; doi:10.3390/genes16101234)
Supplement: Supplementary file 1 [file genes-16-01234-s001.zip › genes-3900835-supplementary.pdf]

Table S1. Physicochemical characteristics of *StCRY*

ProteinID	Name	chromosome	Protein Length	CDSLength (nt)	Molecular Weight (Da)	IsoelectricPoint (pI)	Hydrophobicity (GRAVY)	Localizations
Soltu.DM.04G029750.1	StCRY1	Chr 4	679	2037	76949.08	5.64	-0.49	Nucleus
Soltu.DM.08G018790.1	StCRY2	Chr 8	577	1731	66296.09	9.23	-0.51	Plastid
Soltu.DM.08G025970.1	StCRY3	Chr 8	385	1155	43900.94	7.09	-0.3	Cytoplasm
Soltu.DM.09G023550.1	StCRY4	Chr 9	453	1359	49503.49	8.08	-0.42	Plastid
Soltu.DM.09G026700.1	StCRY5	Chr 9	647	1941	73408.08	5.95	-0.47	Nucleus
Soltu.DM.12G007030.1	StCRY6	Chr 12	583	1749	66149.93	5.23	-0.32	Nucleus
Soltu.DM.12G010860.1	StCRY7	Chr 12	515	1545	58382.39	9.16	-0.32	Nucleus

Table S2. Ka/Ks analysis of *StCRY*

Gene_1	Gene_2	Ka	Ks	Ka_Ks
StCRY1	StCRY2	0.791081156	2.496349641	0.316895175
StCRY1	StCRY3	0.974309155	2.851674603	0.341662108
StCRY1	StCRY4	1.048759055	2.165697549	0.484259243
StCRY1	StCRY6	0.083524151	0.575787909	0.145060619
StCRY1	StCRY7	0.876066978	1.766722937	0.495871175
StCRY2	StCRY4	0.697903565	2.725778817	0.256038223
StCRY2	StCRY7	0.812661932	3.683111219	0.220645504
StCRY3	StCRY4	0.947613351	2.07710476	0.456218371
StCRY4	StCRY5	0.846360882	1.604612687	0.527454936
StCRY4	StCRY6	1.076608039	1.944623582	0.553633129
StCRY4	StCRY7	0.781785945	2.041490782	0.382948555
StCRY5	StCRY7	0.837983824	3.267335362	0.25647316
StCRY6	StCRY7	0.862903401	2.760471545	0.312592753

Table S3. Primers of *StCRY*.

**Name**	**primer-F**	**primer-R**
StCRY1	ATGGGTAGTGGATGAGGGCT	ATGGGTAGTGGATGAGGGCT
StCRY2	CGGATGGGAGCTGAATGGTT	CGGATGGGAGCTGAATGGTT
StCRY3	GTTGTTCAGTCGGGTCGGAT	CCAATACACCACCGGTCCAA
StCRY4	GACGAGCGTCGATTGTTTGG	TAGCCCTATACGGCCCAGTT
StCRY5	TTGCCCGAGCAGTTCATCTT	TCACCGTCCACGTTTGACAT
StCRY6	CTTGTTAGGCTGCCCACTGA	TGCCAGCAGCTTGAAGTACA
StCRY7	TCCTCAAAAGCCGGGTTGAA	CCCTTGAGCACCAACAAACG
B-Actin	GAGGTTCCGTTGCCCAGA	CTGAGCACAATGTTACCATAGAGG
